# Efficient water treatment achieved in recirculating aquaculture system using woodchip denitrification and slow sand filtration

**DOI:** 10.1007/s11356-021-15162-0

**Published:** 2021-07-07

**Authors:** Petra Camilla Lindholm-Lehto, Jani Tapio Pulkkinen, Tapio Kiuru, Juha Koskela, Jouni Vielma

**Affiliations:** Aquatic Production Systems, Natural Resources Institute Finland (Luke), Survontie 9A, FI-40500 Jyväskylä, Finland

**Keywords:** Denitrification, Heavy metals, Off-flavors, Particulate matter removal, Rainbow trout, Recirculating aquaculture system (RAS)

## Abstract

For a recirculating aquaculture system (RAS), a passive water treatment system was designed for efficient discharge nutrient removal and water reuse in RAS production. Denitrification in a woodchip bioreactor filled with birch wood (*Betula pendula*) followed by sand filtration was introduced into a side-loop of an experimental RAS rearing rainbow trout (*Oncorhynchus mykiss*). Denitrification efficiency remained high (96%) throughout the experiment and reached a nitrogen removal rate of 15 g NO_3_-N m^−3^ per day. Sand filtration was used to remove dissolved and particulate matter and improve water quality before being returned to water circulation. To ensure the absence of harmful substances in the system, heavy metals were quantified. Additionally, off-flavor-inducing compounds were quantified in the circulating water and in fish flesh. Significantly higher concentrations of geosmin (GSM) (p<0.05) were observed in the controls compared to side-looped systems, but a similar effect was not observed in the case of 2-methylisoborneol (MIB). Among heavy metals, concentrations of Co (30 μg L^−1^), Ni (40 μg L^−1^), and Pb (140 μg L^−1^) decreased to below 10 μg L^−1^ in the side-loop water after the start-up of the system. Only low concentrations of Cu (5–30 μg L^−1^) were found in the rearing tank water, in both the side-loop and controls. The results indicated that this type of process design is suitable for safely producing fish of high quality.

**Supplementary Information:**

The online version contains supplementary material available at 10.1007/s11356-021-15162-0.

## Introduction

The intensive recirculating aquaculture system (RAS) is a steadily growing type of aquaculture (Blancheton et al. 2007). RASs aim to minimize water requirement which leads to the generation of waste streams high in solids and nutrients. Traditionally, the external water requirement is adjusted, based on the maximum acceptable concentration of nitrate in the RAS (Schuster and Stelz 1998; Martins et al. 2010). The amount of nitrate must be kept at a suitable level (<100 mg L^−1^ NO^3^-N, Chen et al. 2002) to avoid toxic effects for the raised species. Typically, about 5% of the system water or 500–1000 L/kg feed is replaced daily with clean water to prevent the accumulation of nitrate and dissolved organic solids (Masser et al. 1999; Colt 2006; van Rijn et al. 2006). To reach a very low water requirement, nitrogen compounds must be removed via denitrification.

In denitrification, oxidized nitrogen compounds (nitrite, NO_2_^−^, and nitrate, NO_3_^−^) are transformed to elemental gaseous nitrogen (N_2_), typically via heterotrophic microorganisms. Other microbial processes, such as anaerobic ammonium oxidation (Anammox) and chemoautotrophic denitrification can produce N_2_ gas (Burgin and Hamilton 2007). In heterotrophic denitrification, a carbon source is required as an electron donor and for microbial growth (Seitzinger et al. 2006; Tallec et al. 2008). Wood-based material has been used as a carbon source in denitrifying bioreactors since the 1990s (Christianson et al. 2012). Woodchips contain a high C/N ratio, acting as a good carbon source in anoxic conditions (Warneke et al. 2011). In RAS applications, denitrification utilizing woodchips as a carbon source has occasionally been reported in small experimental (Lepine et al. 2020; Lindholm-Lehto et al. 2020), pilot scale (von Ahnen et al. 2019), and full-scale applications (von Ahnen et al. 2018; Lepine et al. 2018).

Denitrification reactors can be used in end-of-pipe applications, but they require a constant input of organic carbon and constant process control, leaving them suitable only for intensive and large-scale systems (van Rijn et al. 2006; von Ahnen et al. 2016). A woodchip bioreactor is a low-tech and cost-effective technology for nitrogen removal with a low maintenance requirement (Lepine et al. 2020), but it has so far only been used for discharge treatment purposes.

Particulate matter needs to be removed in the RAS to avoid excessive microorganisms or blockage of the system. Several pieces of equipment are available for solid removal, including drum filters, swirl separators, and clarifiers to remove particulate from the system, which are designed to treat the total volume of circulating water. In addition, the removed water flow from the system also needs to be treated. In this study, sand infiltration was used to remove particulate matter cost-effectively from the water removed from the system. The infiltration of water through sand removes dissolved and particulate matter and improves water quality. The retention of dissolved organic compounds in the soil proceeds via physical and chemical mechanisms and biological degradation (Wu et al. 2010; Lindroos et al. 2016). These are often utilized in the formation of natural ground water (Lindroos et al. 2016) and the artificial recharge of groundwater (ARG, Peters 1998) for the production of drinking water in the Nordic countries (Kolehmainen et al. 2008).

Intensive water reuse and circulation can increase the accumulation of dissolved metals in RAS (Martins et al. 2011; Davidson et al. 2011). In addition to more conventional sources of metals in typical intensive RASs, a woodchip bioreactor (Werkelin et al. 2005) and sand infiltration are potential sources of metals. Increased concentrations of metals in recirculating water can lead to elevated concentrations in the fish body and flesh (Martins et al. 2009, 2011). For example, Cd, Pb, Ni, and Cr have been found in the gills, liver, and flesh of common carp (*Cyprinus carpio*), ranging from 0.6 to 2.0 μg g^−1^ dry weight (Vinodhini and Narayanan 2008). Deviller et al. (2005) concluded that higher concentrations of metals accumulate in the fish liver and muscles in RAS compared to flow-through systems. Increased levels of As, Pb, Cr, and Mn have also been found in the body, muscles, and liver of Nile tilapia (*Oreocromis nicotilus*, Martins et al. 2011), while Cr, Mn, Co, Ni, Cu, As, Tl, and Cd have been found in the liver and muscles of European seabass (*Dicentrarchus labrax*; Deviller et al. 2005). Additionally, Davidson et al. (2009) suggested that increased concentrations of dissolved metals were likely to cause deformations and higher mortality in rainbow trout (*Oncorhynchus mykiss*). The accumulation of metals in fish flesh is unacceptable, especially when the fish are raised for sale.

Heavy metals can be transported into the fish via feed or directly through the skin and gill epithelium (Staniskiene et al. 2006). Certain elements originate from fish feed at trace levels, such as Fe, Zn, Cu, Co, Mn, Ni, and Se, all essential elements in the normal metabolism of fish (Watanabe et al. 1997; Hertrampf and Piedad-Pascual 2000). Feeds often contain Zn, Cu, Co, and Mn as sulfates (Kaushik 1999). These elements end up in the circulating water via excretion by fish or leaching from feces or unused feed. Typically, higher amounts of metals accumulate in the fish liver than the kidneys or other organs, gills, and flesh (Vinodhini and Narayanan 2008).

Off-flavors perceived in fish are often described as musty and earthy flavors and odors that consumers find objectionable. These off-flavors are mostly caused by geosmin (GSM, trans-1,10-dimethyl-trans-9-decalol) and 2-methylisoborneol (MIB, (1-R-exo)-1,2,7,7-tetramethyl-bicyclo[2.2.1]heptan-2-ol) (Gerber 1968, 1969), although a wide variety of other compounds has been identified (Podduturi et al. 2017; Mahmoud and Buettner 2017). The effects of nitrate levels or denitrification on the development of off-flavors in the RAS has barely been the subject of research so far. Only Schrader et al. (2013) studied the effect of low and high nitrate content on off-flavors in the RAS, but observed no significant effect. Later, Azaria et al. (2020) observed that among *Betaproteobacteria* genera, *Thauera* utilized terpenes to fuel denitrification as well as *Comamonas* and could utilize GSM and MIB as a carbon and energy source. As we were using a woodchip denitrification step, the formation of off-flavors was of interest in this study. The concentrations were analyzed both in circulating water and in fish flesh to determine if the woodchip denitrification showed any difference in the formation and accumulation of off-flavors.

In our previous study (Lindholm-Lehto et al. 2020), we showed that a passive water treatment loop with denitrification in a woodchip bioreactor followed by sand infiltration was a suitable treatment for regenerating system effluent into intake water. In this study, the aim was to identify the optimal process design to achieve a stable nitrogen removal efficiency for a longer period. Second, we wanted to study if the denitrification affected the accumulation of the most common off-flavor compounds. The third goal was to identify and quantify the accumulation of heavy metals into the circulating water and fish flesh that should raise any concern.

## Materials and methods

### Experimental setup

The experiment was performed in an experimental RAS platform at the Natural Resources Institute Finland (Luke) Laukaa fish farm. The full description of the experimental RAS facility was reported by Pulkkinen et al. (2018). A passive water treatment system was connected to a randomly selected, individual RAS. Additionally, control systems without the passive treatment loop were operated accordingly (Table 1). The experiment was conducted by using randomly allocated duplicate systems. In total, four individual RASs were used which contained components described in detail in Pulkkinen et al. (2018) and in Lindholm-Lehto et al. (2020).

**Table 1:** Operational design of the RAS units: side-loops (n=2), controls (n=2), and rearing conditions of rainbow trout (*Oncorhynchus mykiss*) in the experiment

Characteristics	Value	Unit
Water renewal		
Side-loop	100	L kg feed^−1^ d^−1^
Control	500	
Side-loop	48	L d^−1^
Fish quantity per tank	55→18*	pcs
Fish mortality*		
Side-loop	13	pcs
Control	29	
Fish density		
Initial-final	16.4→30.9	kg m^−^^3^
Average fish weight**	148.6→705.3	g
Average feed per day	88	g d^−1^
Feed pellet size	3→5	mm

*Includes mortalities and removed individuals

**Average fish weight and weight gain during the experiment

**Fig. 1:**
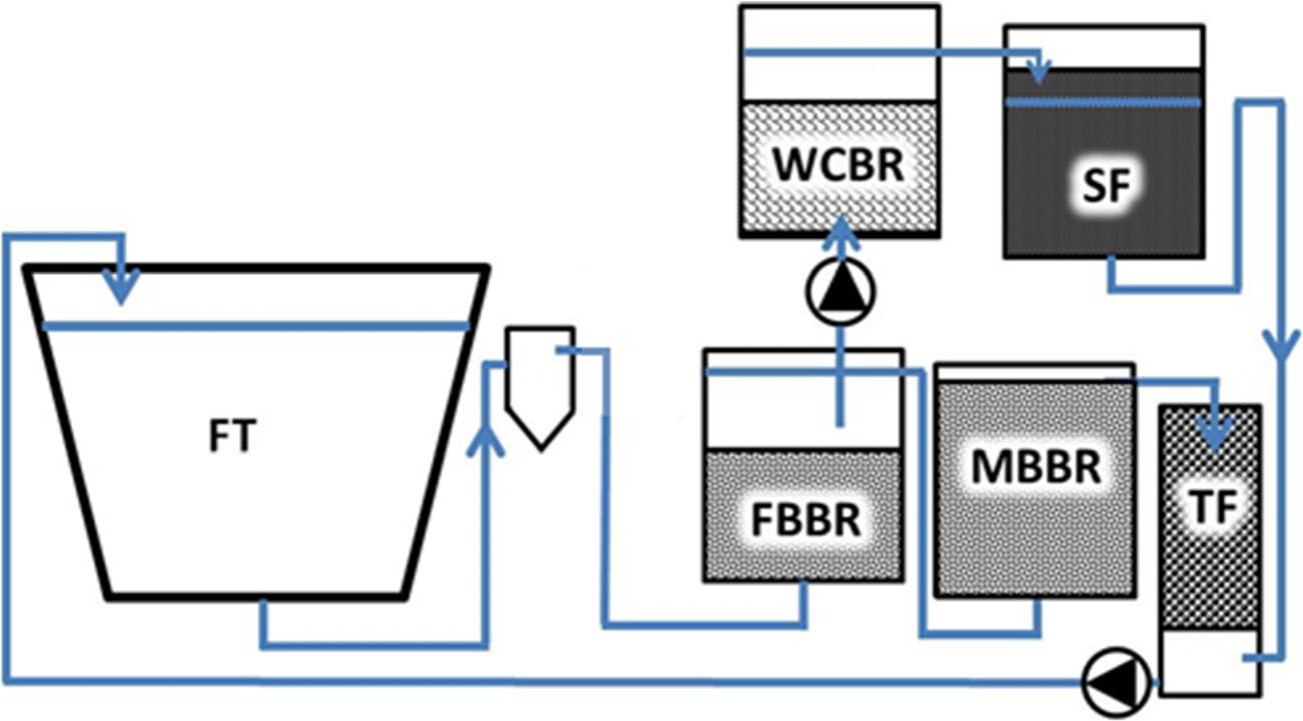
A flowchart of the experimental setup: a fish tank (FT), swirl separator (SS), fixed-bed reactor (FBBR), moving bed reactor (MBBR), trickling filter (TF), a side-loop with a woodchip bioreactor (WCBR), and a sand filter (SF).

A passive water treatment side-loop (48 L d^−1^) consisted of a woodchip bioreactor filled with 125 L of fresh unbarked silver birch (*Betula pendula*) woodchips (<5 cm, effective porosity n_e_ 0.65), aiming for 87% denitrification efficiency (2.5 g NO_3_-N d^−1^), with an HRT (hydraulic retention time) of 1.7 days. The denitrification efficiency of 87% was selected by aiming for as high NO_3_-N concentration in the tank water of the side-looped system as in the controls. A sand filter (160 L, 55 cm in height) with an effective porosity (n_e_) of 0.35 was packed with an 80% saturation zone (44 cm) and an HRT of 1.1 days before returning the water to the pump sump. The water flowed passively first through the woodchip bed and then through the sand filter, exiting the reactor via an overflow (Fig. 1). Denitrification efficiencies were calculated after each step of the side-loop at the beginning and end of the experiment by measuring NO_3_-N (LCK340, DS 3900, Hach, Loveland, USA).

Clean replacement water was taken from the oligotrophic Lake Peurunka (area 694 ha, 59 600 m^3^) and used at a relative water renewal rate of 12 L d^−1^ (100 L kg^−1^ feed d^−1^) in the side-looped systems and 60 L d^−1^ (500 L kg^−1^ feed d^−1^) in the control systems.

Oxygen saturation was kept above 80% in the fish tanks by injecting oxygen into the pump sumps, and CO_2_ content was maintained below 15 mg L^−1^. The water temperature was maintained at 15.5 ± 0.7 °C and the pH at 7.2 throughout the experiment by adding NaOH (20% aqueous, Finnish chemicals Oy, Joutseno, Finland) solution. The circulating water flow rate was set to 0.2 L s^−1^. Nitrite and nitrate nitrogen, total suspended solids, total organic carbon, and turbidity were measured by an optical spectro::lyser sensor (S::can, Vienna, Austria), oxygen (OxyGuard, Farum, Denmark), CO_2_ (Franatech, Lüneburg, Germany), and pH (ProMinent, Heidelberg, Germany) online every 6 min from the rearing tanks to monitor the water quality. Additionally, the total ammonia, nitrite, and nitrate nitrogen were monitored weekly by quick spectrophotometric laboratory tests (Procedure 8038 Nessler, LCK340, LCK341, UN3316 9 II, Supplementary table S1). Alkalinity was monitored by a standard titration method (ISO 9963−1:1994, TitraLab AT1000, Hach, Loveland, USA), and turbidity was determined with a Hach DR 3900 Turbidimeter, USA. Removal of NO_3_-N was calculated based on the results of the quick spectrophotometric tests before and after the woodchip bioreactor and the sand filter, the water flow in the side-loop (48 L d^−1^), and the volume of woodchips (0.125 m^3^).

### Fish and feeding

The experiment was started in the spring of 2019 and continued for 7 months (30 weeks). Before starting the passive water treatment loop, fish were allowed to acclimate to the experimental units for 5 weeks. The experiment was started with 55 fish in each tank, weighing on average 148.6 ± 10.7 g (16.4 kg m^−3^) and increasing in weight to 705 ± 120 g (30.9 kg m^−3^) during the experiment (Table 1). The fish were fed similar amounts to each system with BioMar (Orbit 9030, Aarhus, Denmark) 3 mm and Raisioaqua Circuit Red (Finland) 5 mm pellets. The main ingredients in Raisioaqua consisted of fish meal made of Baltic herring and sprat, soya meal, and horse bean, including a total of 0.95–1.15% P and 7.52–7.84% N, as given by the manufacturer. In BioMar, there was 44–47% protein, 28–31% crude lipid, 10.4–16.4% carbohydrates, and 0.9% P, also given by the manufacturer. The fish were fed 9 times per day by a computer-controlled feeding system (Arvotech, Joroinen, Finland).

There were four intermediate weighings to monitor the tank biomass and number of fish and to adjust the feeding correctly. The fish were visually inspected on a daily basis, and any mortalities were removed and recorded.

### Sample collection

The circulating water was collected approximately once a month, except for once a week in the first 3 weeks. In total, aqueous samples were taken nine times directly from the rearing tank, after the woodchip bioreactor and after the sand filter. For the chemical analyses, water samples were collected in new 250 mL high-density polyethylene (HDPE) plastic jars with HDPE plastic caps. Samples were stored frozen at −22 °C, except for the samples for the elemental analyses, which were stored at +2 °C.

Fish were sampled in the beginning of August and in the end of September. In both cases, five fish were selected from each rearing tank and humanely euthanized. Each fish was weighed, gutted, and stored at −22 °C before the analyses. Pooled samples of the five individuals were used for the off-flavor and elemental analyses. The dry matter content of fish flesh was determined by the ISO 638:2008 standard method.

### Chemical analyses

#### Off-flavors

Off-flavors, more specifically geosmin (GSM, trans-1,10-dimethyl-trans-9-decalol) and 2-methylisoborneol (MIB, 1-R-exo-1,2,7,7-tetramethyl-bicyclo[2.2.1]heptan-2-ol) were analyzed from the aqueous samples and fish flesh. In short, GSM and MIB were analyzed by a headspace solid-phase microextraction (HS-SPME), followed by gas chromatography mass spectrometry (GC-MS). 1 mL of aqueous or 1 g of fresh sample was placed in a 10-mL HS vial with 750 μL of saturated NaCl solution (aq, Merck, 98%). 30 μL of internal standard (2-isobutyl-3-methoxypyrazine, IBMP, 99%) from Merck was diluted in methanol (J.T. Baker (≥99.8%)), added to a vial, and closed with polytetrafluoroethylene (PTFE) septum caps (Merck).

SPME was performed with a manual assembly, and the analytes were adsorbed onto an extraction fiber coated with divinylbenzene/carboxene/polydimethyl siloxane (DVB/CAR/PDMS, 1 cm, 50/30 μm, part no. 57328-U) from Supelco, Merck. The sealed vial was placed in a water bath at 60 °C, and the fiber was exposed in the headspace for 30 min. The fiber was then directly introduced into the GC-MS injection port for desorption.

A GC-MS analysis was performed with an Agilent 6890 series/5973N GC/MSD (Palo Alto, CA, USA) system with a Phenomenex Zebron ZB-5MSi (Torrance, CA, USA) capillary column (30 m × 0.25 mm × 0.25 μm). The injector was adjusted at 270 °C in the splitless mode, followed by the carrier gas (helium) at a flow rate of 0.7 mL min^−1^. The oven temperature started at 45 °C for 3 min increasing 28 °C min^−1^ up to 300 °C in 14.7 min. Selected ion monitoring (SIM) was used for the detection of GSM, MIB, and the internal standard IBMP with *m/z* 112, 126, 182 (GSM), *m/z* 95, 135, 168 (MIB), and *m/z* 137 (IBMP). Peak areas of the internal standard and analytes were used to quantify GSM and MIB. The detailed method description and method validation have been reported in Lindholm-Lehto et al. (2019). For aqueous samples, the levels of quantification (LOQ) were 2.8 ng L^−1^ (GSM) and 1.6 ng L^−1^ (MIB), while for fish, they were 2.1 ng g^−1^ (GSM) and 1.5 ng g^−1^ (MIB).

#### Anions

Selected anions, including chloride (Cl^−^), nitrite (NO_2_^−^), nitrate (NO_3_^−^), sulfate (SO_4_^3−^), and phosphate (PO_3_^4−^) were monitored in the circulating water throughout the experiment. The method based on ion chromatography has been reported in detail by Lindholm-Lehto et al. (2020). Before the analysis, the samples were first purified by running them through a solid-phase extraction (SPE) cartridge (Phenomenex Strata® C18-E, 500 mg/3 mL, 55 μm, and 70 Å) and filtered through a 0.2-μm syringe filter (13 mm Ø, regenerated cellulose, Teknokroma).

The chromatographic separation was conducted on a Dionex DX-500 ion chromatography equipment (Dionex, Sunnyvale, CA, USA) with an anion pre-column (Ion Pac^TM^ AG11-HC-4 μm, 4 mm × 25 mm), an anion separation column (Ion Pac^TM^ AS11-HC-4 μm, 4 mm × 250 mm), an anion self-generating suppressor (ASRS 600, 4 mm), and a conductivity detector (CD20). Elution was performed at a flow rate of 1.0 mL min^−1^ with a linear gradient from 14 mM KOH for 5 min to 60 mM KOH over the course of 12 min. After 4 min at 60 mM, the concentration was decreased again to 14 mM, taking a total of 26 min. The inlet pressure was about 2000 psi, the column temperature 30 °C, and the sample injection volume 25 μL. A suppressor current of 149 mA was chosen for the conductivity detector.

Standard solutions of 5 mg L^−1^ for sodium chloride (NaCl), sodium nitrite (NaNO_2_), sodium nitrate (NaNO_3_), disodium hydrogen phosphate (Na_2_HPO_4_), and 10 mg L^−1^ sodium sulfate (Na_2_SO_4_) were prepared by diluting an accurate amount of pure compound (≥ 99 %, Merck) in UHQ water (internal resistance ≥18.2 Ω at 25 °C) by Millipore (Bedford, MA, USA) and filtered through a 0.2-μm syringe filter. The applied method was validated as reported by Lindholm-Lehto et al. (2020). The levels of detection (LOD) ranged from 0.093 to 1.0 mg L^−1^ and LOQs from 0.102 to 1.16 mg L^−1^, all with good linearities (0.9973–0.9996, Supplementary Table S2). The equations of linearity analysis were used for the quantification of sample concentrations.

#### Fatty acids

The sample pH was first adjusted to below 3 with a few drops of 1M HCl (aq) to ensure the acidic form of fatty acids if present. 4 mL of sample was added in a screw-capped Kimax-tube for liquid-liquid extraction (LLE) with 2 mL of methyl *tert*-butyl ether (MTBE, ≥99.8%, Merck, Darmstadt, Germany). The solution was stirred thoroughly, centrifuged at 300 g for 5 min (centrifuge 1.0), and the clear supernatant was collected. Heneicosanoic acid (30 μL, 95 μg mL^−1^ in MTBE, and purity ≥99%, Merck, Saint Louis, MO, USA) was used as an internal standard. The extraction procedure was repeated three times, and the samples were prepared as triplicates. The extracts were evaporated to dryness under a gentle stream of nitrogen. For derivatization to trimethylsilyl esters, 760 μL of pyridine (dried with KOH granules, Merck, max. 0.002 % Na) and 330 μL of 25% *o*-bis-(trimethylsilyl)-trifluoroacetamide (BSTFA) with 1% trimethyl chlorosiloxane (TMCS, Alfa Aesar, Heysham, Lancashire, UK) were added to the evaporation residue. The solution was heated in an oven at 70 °C for 1 h.

Gas chromatography with flame ionization detection (GC-FID) analysis was performed with a Shimadzu GC-2010/FID instrument, equipped with a ZB-5MSi column (7HG-G018-11, 30 m × 0.25 mm × 0.25 μm) and an autosampler (AOC-20i). The oven temperature was held at 70 °C for 1 min, heated to 250 °C over the course of 10 min, then to 300 °C in 5 min, and held for another 5 min. The FID was operated at 300 °C with a sampling rate of 40 msec, a helium flow of 40 mL min^−1^, and an air flow of 400 mL min^−1^. Injections of 1 μL per sample were made in the splitless mode.

The compounds were identified using an Agilent 6890 series/5973 N GC/MSD (Palo Alto, CA, USA) system with a mass spectrometric detector under elector ionization (70 eV) and a Phenomenex Zebron ZB-5MSi (Torrance, CA, USA) capillary column (30 m × 0.25 mm × 0.25 μm) with a similar oven temperature program to the GC-FID equipment. For the identification of the chromatogram peaks, the appropriate interpretation of the mass spectra was used, based on the National Institute of Standards and Technology [NIST] mass spectral library. LOD and LOQ were calculated for the standard solution, resulting in 0.12 mg L^−1^ (LOD) and 0.16 mg L^−1^ (LOQ). A more detailed method description and validation data have been reported in Lindholm-Lehto et al. (2020).

### Elemental analyses

#### Sample digestion and elemental analyses

A microwave acidic digestion of the circulating water was performed according to US EPA 2007a, method 3015. For practical reasons, the volume of the sample was reduced by half to 18 mL. 3 mL of HNO_3_ (65%, Fluka) was added, and the sample was placed into a polytetrafluoroethylene (PTFE) tube. The tubes were capped and heated in a CEM Mars 6 (Hosmed) microwave oven to 170 °C over the course of 10 min and held for another 10 min at 170 °C (US EPA 2007a, method 3015). For solid samples, their moisture content was determined (ISO 638:2008), and 0.5 g of sample (calculated as dry) was prepared according to US EPA 2007a method “animal tissue (dry).” Then, 8.70 mL of HNO_3_ (69 %, Fluka) was added. The samples were left to stand for 15 min before heated to 200 °C over the course of 15 min and held for another 15 min at 200 °C in a CEM Mars 6 (Hosmed) microwave oven. Both aqueous and solid samples were left to cool to 30 °C, transferred into a 40-mL flask and brought to volume with UHQ water.

The quality assurance of the digestion method was achieved by performing the analysis of spiked samples and method blanks. The samples were gravimetrically prepared in 1% HNO_3_ (w/w) prior to inductively coupled plasma mass spectrometry (ICP-MS) analysis. Samples were prepared and analyzed in duplicate, with the recoveries ranging from 94 to 105%.

Measurements of selected elements were performed with a quadrupole-based Perkin Elmer NexION® 350 D ICP-MS system with an octapole collision cell and baffled cyclone electrospray ionization (ESI) cooled to +2 °C. The operating conditions and specifications were listed in Supplementary Table S3. Before use, the ICP-MS was tuned with a 1 μg L^−1^ tuning solution (Perkin Elmer NexION Setup Solution N8145051). A standard solution of the selected trace elements (Cd, Co, Cu, Mn, Ni, and Pb) was prepared at a concentration of 100 μg L^−1^ (1% NHO_3_, w/w), while an internal standard solution (Bi, In, Ga, and Ge, 100 μg L^−1^ in 1% NHO_3_, w/w) was used as a reference and added via a mixing T-piece. All solutions were gravimetrically prepared in 1% HNO_3_, w/w. As previously mentioned, LODs, LOQs, and linearities (R^2^) were determined for the selected elements (Supplementary Table S4). The full method description and the method validation have been described in more detail in Lindholm-Lehto et al. (2020).

Selected major and minor elements were analyzed with a Perkin-Elmer (Optima 8300, Norwalk, CT, USA) inductively coupled plasma optical emission spectrometer (ICP-OES), equipped with a glass concentric nebulizer and a cyclonic spray chamber. The plasma was viewed axially for potassium (K), phosphorous (P), sulfur (S), and zinc (Zn) and radially for aluminum (Al), calcium (Ca), iron (Fe), and magnesium (Mg). The analytical parameters of the instrument were as follows: RF power 1.5 kW, plasma gas flow rate 8 L min^−1^, auxiliary gas flow rate 0.2 L min^−1^, nebulizer gas flow rate 0.6 L min^−1^, rinse time 10-15 s, and sample uptake 1.5 mL min^−1^. The measurements were performed in 5% HNO_3_. An external calibration was used by preparing 0.5, 1, 10, 30, and 60 mg L^−1^ standard solutions, containing Al, Ca, Fe, K, Mg, P, S, and Zn. All reagents were of analytical grade. The accepted relative standard deviation of three replicate measurements was less than 20%, with an external calibration. The optimal analytical wavelengths for the measurements were (nm) as follows: Al (396.153), Ca (315.887), Fe (238.204), K (766.490), Mg (279.077), P (177.50), S (182.563), and Zn (206.2). The LODs, LOQs, and linearities were listed in Supplementary Table S5.

### Statistical analyses

Statistical analyses of elements and off-flavors were performed with IBM SPSS Statistics for Windows, Version 26.0 (Armonk, NY: IBM Corporation, released 2019). The differences in the mean values in the experiment and in the controls were tested by paired samples t-tests. The confidence interval was set at 95%.

The average feed conversion ratio (FCR = feed intake/growth), specific growth rate (SGR = (ln (initial weight)-ln (end weight))/feeding days × 100), and feeding per day were calculated from the 5 stages of the intermediate weighings. The differences between the side-looped systems and controls on growth, feed intake, feed conversion ratio, specific growth rate, and mortality were analyzed with one-way analysis of variance (ANOVA) using the IBM SPSS software.

## Results

### Fish and feeding

Feed conversion ratios and specific growth rates in the systems with the woodchip bioreactor and the sand filter side-loop were similar to those in the controls. Lower mortality was observed in the side-looped systems than in controls (Table 2), but no significant difference (p < 0.05) was found between the side-looped systems and controls, excluding SGR (Table 3). No signs of unusual behavior or signs of stress or discomfort in fish were observed.

**Table 2:** Initial and final biomass, growth, and mortality (n=2, ± SD) in the side-looped systems and controls, including a woodchip bioreactor and a sand filter. The feed conversion ratio (FCR), specific growth rate (SGR), and feed per day (n=2, ± SD) were calculated as averages of the 5 stages between the intermediate weighings

	Control*	Treatment
Initial biomass (g)	8140 ± 33	8200± 33
Final biomass (g)	15,200 ± 688	15,700 ± 150
Growth (g)	19,300 ± 680	17,900 ± 250
Feed (g d^−1^)	86 ± 0.6	86 ± 0.5
FCR	1.09 ± 0.02	1.16 ± 0.05
SGR (% d^−1^)	0.66 ± 0.02	0.56 ± 0.02
Mortality (%)*	28 ± 10	11 ± 1

*Mortalities and removed individuals were considered

**Table 3:** Differences between the side-looped systems and controls (n=2) on growth, feed intake, feed conversion ratio (FCR), specific growth rate (SGR), and mortality with one-way ANOVA.

ANOVA	Sum of squares	Mean square	F value	Significance
Growth (g)	1,762,256	1,762,256	3.349	0.209
Feed (g d^−1^)	0.090	0.090	0.148	0.738
FCR	0.005	0.005	1.849	0.307
SGR (% d^−1^)	0.009	0.009	14.440	0.063
Mortality (%)	0.027	0.027	2.663	0.244

### Nitrate removal

At the beginning of the experiment, the nitrate removal efficiency was 92% in the woodchip bioreactor, and the average nitrate removal rate was 11 g NO_3_-N m^−3^ of woodchips d^−1^. A removal efficiency of another 26% was achieved in the sand filter, resulting in a total efficiency of 98%. After a month of the experiment, the nitrate removal efficiency increased to 96% after the woodchip bioreactor and another 17% in the sand filter, yielding a total efficiency of 99%. At the end of the experiment, the average nitrate removal rate increased to 15 g NO_3_-N m^−3^ of woodchips d^−1^.

### Off-flavors

**Fig. 2:**
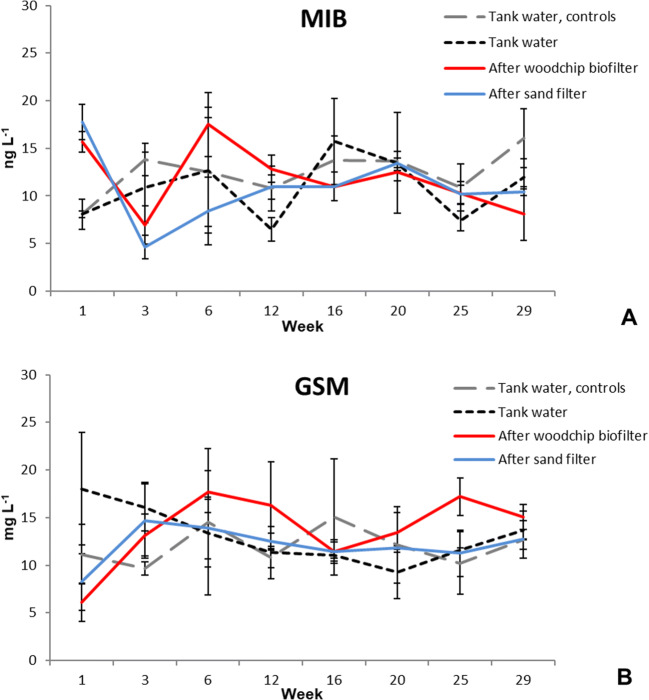
Concentrations of MIB (**A**) and GSM (**B**) in the tank water with the side-looped systems, controls, in the woodchip bioreactor, and in the sand filter during the 30 weeks of the experiment (ng L^−1^, ± SD, n=4).

The concentrations of MIB in the circulating water ranged from 6.5 to 16 ng L^−1^ (Fig. 2A), while those of GSM ranged from 6.1 to 18 ng L^−1^ (Fig. 2B). The concentrations remained relatively stable throughout the experiment, but in some occasions, the concentrations of GSM were higher than those of MIB. In the case of circulating water, no significant difference (p<0.05) between the treatment and controls was observed (Supplementary Table S6).

**Fig. 3:**
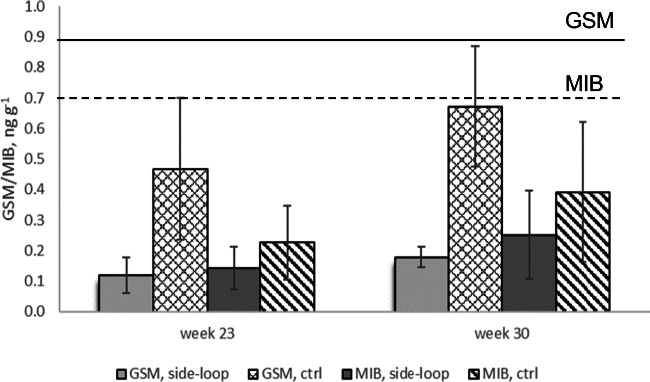
Concentrations of MIB and GSM in the fish fillet after 23 and 30 weeks of experiment (ng g^−1^, ± SD, n=4). Sensory detection thresholds for rainbow trout have been marked: 0.9 ng g^-1^ for GSM, line and 0.7 ng g^−1^ for MIB, dashed line (Robertson et al. 2005)

In the fish fillet, the levels of GSM were higher than those of MIB. They were higher in the side-looped systems than in controls with significant difference (p<0.05), but for MIB, no significant difference (p<0.05) was observed (Supplementary Table S6). Overall, very low concentrations were detected, remaining below the limits of the sensory threshold (Fig. 3).

### Anions

**Fig. 4:**
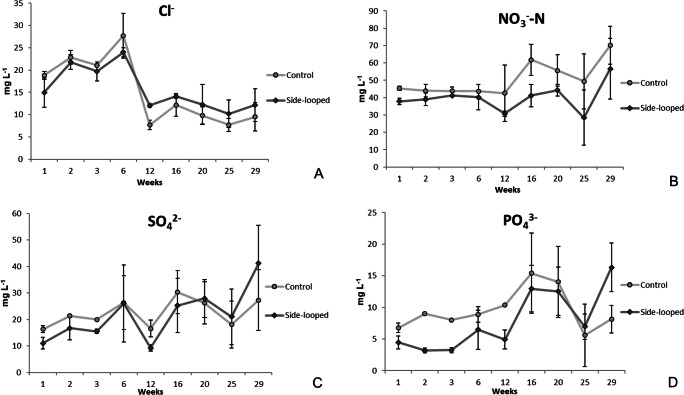
Concentrations of chlorine (Cl^−^, **A**), nitrate-N (NO_3_-N, **B**), sulfate (SO_4_^2−^, **C**), and phosphate (PO_4_^3−^, **D**) in the rearing tank water during the 30 weeks of the experiment (mg L^−1^, ± SD, n=4). In the side-looped system, part of the circulating water was led though a woodchip bioreactor, and a sand filter for nitrate and particulate matter removal. Controls were run without a side-loop.

The concentrations of chlorine were measured from the rearing tank water, and the circulating water in the woodchip bioreactor, and in the sand filter (Fig. 4A, Supplementary Fig. S1A). In all these locations, the concentrations were about 20 mg L^−1^. Apart from a single increase to about 28 mg L^−1^ after 6 weeks of the experiment, the concentrations dropped and remained in the range of 10–15 mg L^−1^ levels until the end of the experiment.

The concentrations of nitrate, calculated as NO_3_-N, remained very stable during the first 12 weeks in the rearing tank water of both system types (Fig. 4B). In the latter part of the experiment, the concentrations fluctuated somewhat but remained at moderate levels (below 70 mg L^−1^ NO_3_-N). In the case of the side-looped systems, no nitrate was observed during the first half of the experiment (Supplementary Fig. 1B). Later, low levels (below 15 mg L^−1^ NO_3_-N) were detected after the woodchip bioreactor and sand filter. Furthermore, the concentrations were higher in the controls than those with the side-loop. This is in agreement with the aim of nitrate removal by denitrification.

The concentrations of sulfate remained in a range of 20–30 mg L^−1^ throughout the experiment (Fig. 4C). Only at the end of the experiment was an increase to 40 mg L^−1^ observed. The concentrations were very similar in systems with and without the side-loop. Levels of phosphate ranged from 5 to 15 mg L^−1^, but were mostly higher in the systems without the side-loop (Fig. 4D). Concentrations of a similar range were observed in the side-looped system (Supplementary Fig. S1).

### Fatty acids

**Fig. 5:**
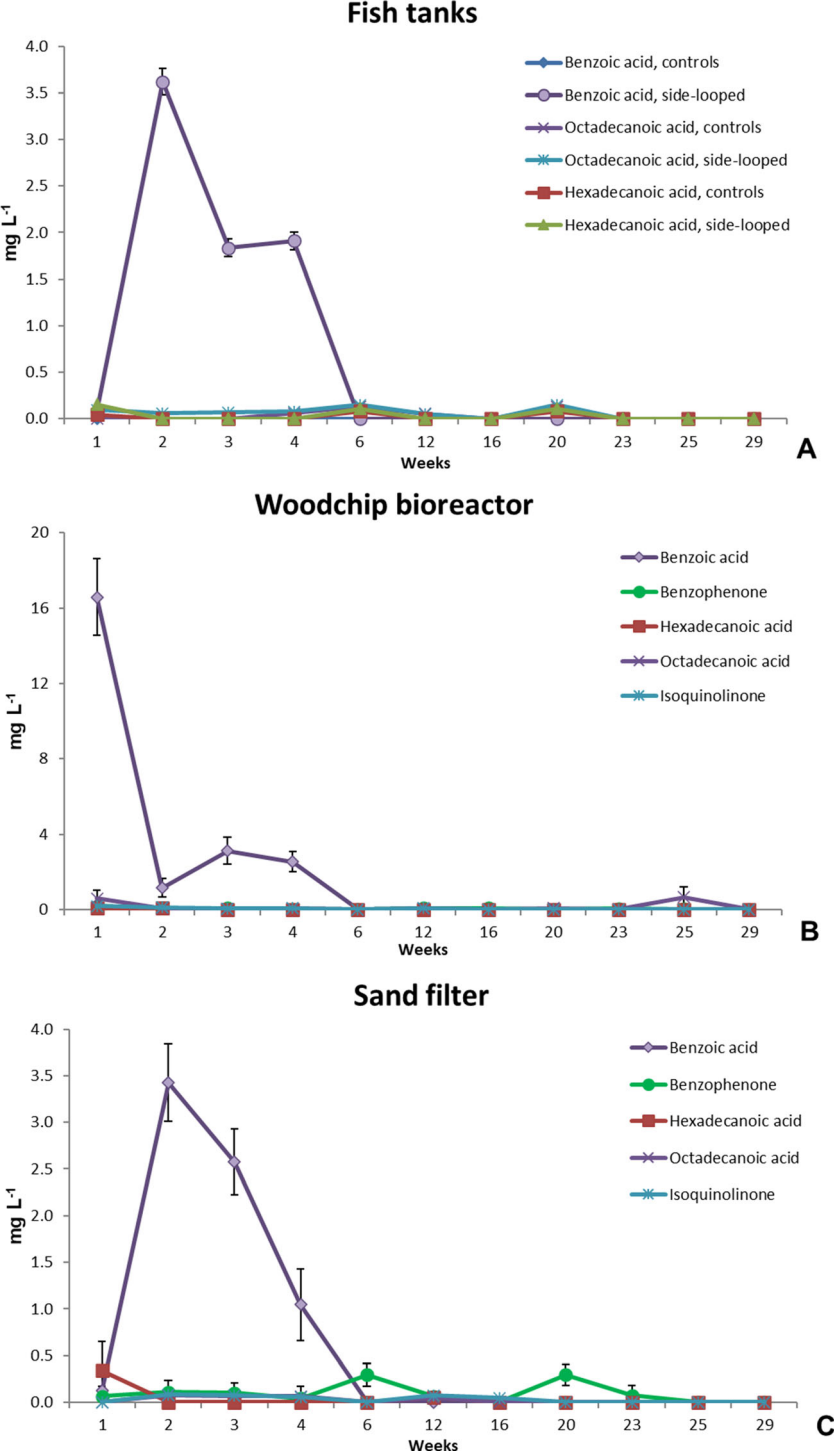
Concentrations of benzoic acid, benzophenone, hexadecanoic acid, octadecanoic acid, and isoquinolinone in the rearing tank water (**A**), the circulating water in the woodchip bioreactor (**B**), and the sand filter (**C**) during the 30 weeks of the experiment (mg L^−1^, ± SD, n=6).

Long-chained fatty acids and other organic compounds were found in the rearing tank water (Fig. 5A) and in the side-loop (Fig. 5B, C). Benzoic acid was found up to 3.6 mg L^−1^ in the rearing tank water of the side-looped systems, but not in the controls. Benzoic acid was found up to 16 mg L^−1^ in the woodchip bioreactor and 3.0 mg L^−1^ in the sand filter. The highest concentrations were found at the beginning of the experiment, but decreased rapidly either to a very low level or below the LOD.

### Elemental analysis

**Fig. 6:**
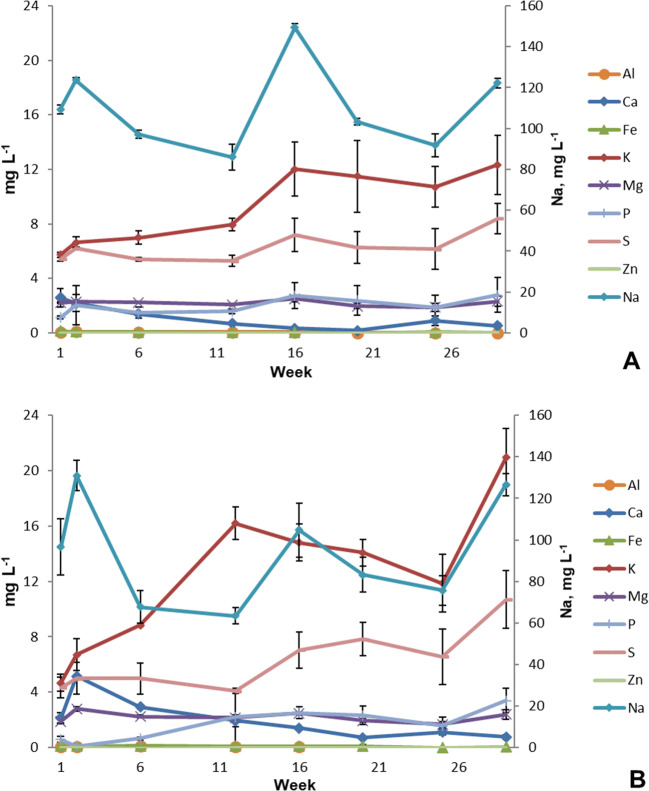
Concentrations of aluminum (Al), calcium (Ca), iron (Fe), potassium (K), magnesium (Mg), phosphorous (P), sulfur (S), zinc (Zn), and sodium (Na, on the right hand side) in the tank water of the controls (**A**) and the side-looped systems (**B**) during the 30 weeks of the experiment (mg L^−1^ ± SD, n=4).

In the experiment and controls, the concentrations of selected elements were very stable throughout the experiment in the tank water (Fig. 6) and in the side-loop (Supplementary Fig. S2). In the tank water, the concentrations of K increased from 4.3 to 20.9 mg L^−1^ in the side-looped systems (Fig. 6B) and from 5.7 to 12.3 mg L^−1^ in the controls (Fig. 6A). Additionally, the concentrations of S increased slightly (4.3–10.7 mg L^−1^ and 5.4–8.4 mg L^−1^ in the controls) in both the side-looped systems and controls. The concentrations of Al, Mg, Fe, and P remained low, below 3 mg L^−1^ throughout the experiment. Those of Ca remained below 5 mg L^−1^. Based on the statistical analysis, the concentrations of Ca, Cu, Fe, K, and Na showed a significant difference (p<0.05), while the other elements did not (Supplementary Table S7). Zn was not detected in the rearing tank water, but at a low range of μg L^−1^ was found in the woodchip bioreactor (Supplementary Fig. S2A). Generally, the concentrations of all the selected elements were lower in the sand filter (Supplementary Fig. S2B) than those in the woodchip bioreactor (Supplementary Fig. S2A), apart from the high concentrations of Al at the beginning of the experiment.

In the tank water, the concentrations of Cd, Co, Mn, Ni, and Pb remained below the LODs. Only small concentrations of Cu were detected, ranging from 5.7 to 22.2 μg L^−1^, while they ranged from 9.3 to 30.2 μg L^−1^ in the controls. However, the concentrations between the side-looped systems and controls showed significant differences (p<0.05, Supplementary Table S7). In feed, the selected trace elements ranged from 4.3 to 7.0 μg g^−1^ dw Cu (Table 4).

**Table 4:** Concentrations of cadmium (Cd), cobalt (Co), copper (Cu), lead (Pb), manganese (Mn), and nickel (Ni) in fish flesh (± SD, n=4) of side-looped systems and controls and fish feed (Raisioaqua Circuit Red; BioMar Orbit 9030, ± SD, n=2), both expressed as μg g^−1^ (dry)

Fish/feed	Cd (μg g^−1^)	Co (μg g^−1^)	Cu (μg g^−1^)	Pb (μg g^−1^)	Mn (μg g^−1^)	Ni (μg g^−1^)
Fish controls, week 23	0.001±0.0	0.06±0.02	1.16±0.20	0.048±0.02	0.70±0.24	0.54±0.13
Fish, week 23	0.0±0.0	0.06±0.02	1.04±0.19	0.022±0.01	0.89±0.32	0.46±0.13
Fish controls, week 30	0,001±0.0	0,02±0.00	1,29±0.40	0.030±0.01	0,81±0.50	0,43±0.40
Fish, week 30	0,004±0.0	0,02±0.01	0,92±0.17	0.029±0.01	0,48±0.03	0,27±0.10
Circuit red	0.043±0.0	0.20±0.04	4.85±0.01	0.068±0.00	83.98±6.0	0.94±0.02
Orbit 9030	0.064±0.0	0.16±0.00	7.62±0.06	0.056±0.00	29.95±4.4	0.96±0.00

**Fig. 7:**
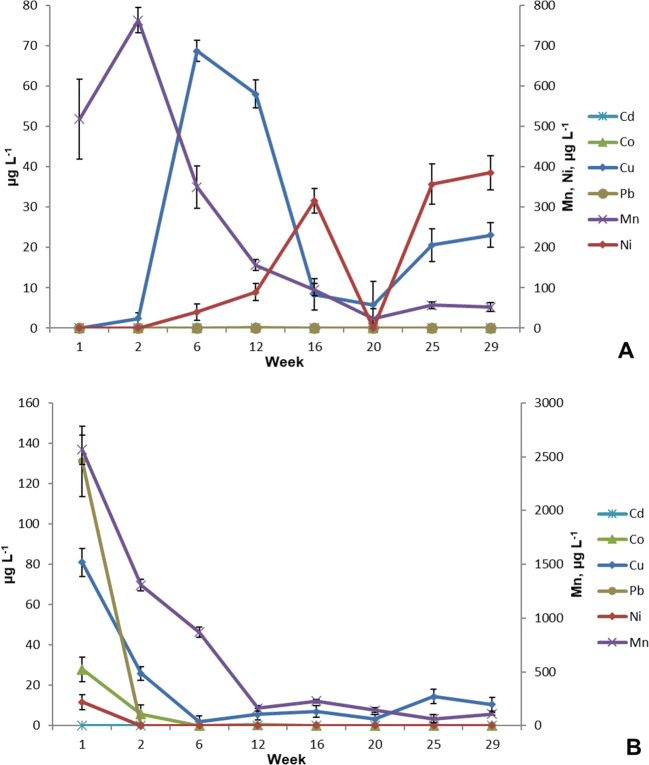
Concentrations of cadmium (Cd), cobalt (Co), copper (Cu), lead (Pb), manganese (Mn), and nickel (Ni) (Mn and Ni, axis on the right-hand side) in the circulating water in the woodchip bioreactor (**A**) and in the sand filter (**B**) during the 30 weeks of the experiment (μg L^−1^, ± SD, n=4).

After the woodchip bioreactor, the concentrations of Mg increased to 763 μg L^−1^ at the beginning of the experiment, but then decreased to about 50 μg L^−1^ (Fig. 7). Ni increased to 385 μg L^−1^ during the experiment. On the other hand, Cu increased to 70 μg L^−1^, but mostly ranged from 10 to 25 μg L^−1^. Apart from one sampling point, Pb, Cd, and Co remained below the LODs.

The highest concentrations of the selected trace elements were detected in the sand filter during the first week of the experiment (Fig. 7B), but they rapidly decreased to below 20 mg L^−1^, and in the case of Mn to 100 mg L^−1^. Cd, Co, Ni, and Pb were detected only on a few occasions at the beginning of the experiment.

## Discussion

### Bioreactor efficiency

Fish grew similarly in both types of RAS used in the experiment with no significant difference (p<0.05, Table 3). However, a 15% higher specific growth rate was observed in the control RAS. The feeding rate was limited (approx. 0.5% d^−1^), although we did not expect major effects on growth parameters. During the experiment, high nitrate removal (up to 99% in total) and removal rates of 11–15 g NO_3_-N m^−3^ woodchips d^−1^ were achieved. Overall, a wide range of nitrate removal rates (2–22 g NO_3_-N m^−3^ d^−1^) by woodchip bioreactors has been reported (Schipper et al. 2010; Christianson et al. 2012; von Ahnen et al. 2016). For example, Greenan et al. (2006) reported denitrification of 19–26 g NO_3_-N m^−3^ d^−1^ by woodchip bioreactors when treating water with 10–80 mg NO_3_-N L^−1^. Later, Greenan et al. (2009) achieved a 50% reduction from 50 g NO_3_-N m^-3^ in 2.8 days at 10 °C, while von Ahnen et al. (2018) achieved 16.4–16.8 g NO_3_-N m^−3^ d^−1^.

A longer hydraulic retention time (HRT) typically results in higher removal efficiency (%), while a shorter HRT yields a higher removal rate (g NO_3_-N m^−3^) (Lepine et al. 2016). In this study, the HRT was increased from our previous experiment’s HRT of 1.5 (1.4–2.3 g NO_3_-N d^−1^ in 1.5 days, Lindholm-Lehto et al. 2020) to an HRT of 1.7 (2.5 g NO_3_-N d^−1^ in 1.7 days). Compared to the previous results, higher nitrate removal efficiencies were achieved even at the end of the experiment, suggesting an improved dimensioning of the system.

In this study, only nitrate concentrations were monitored from the inlets and outlets of the reactors, leaving the proportions of nitrogen end-products unconfirmed and a subject for further research. The nitrate removal rate can decrease by up to 50% during the first year of operation (Robertson 2010). In this experiment, the removal rate remained constant, although the experiment was designed to last for only 7 months, leaving more long-term observations to future experiments.

### Fatty acids

Woodchip bioreactors filled with a variety of wood species have been studied, such as white ash (*Fraxinus americana*), Norway maple (*Acer platanoides*) (Lepine et al. 2020), a combination of spruce (*Picea* sp.), poplar (*Populus* sp.), beech (*Fagus* sp.) (von Ahnen et al. 2018), and a less specific hardwood blend (Lepine et al. 2016). In RAS applications, any trace compounds of woodchip-origin that are harmful or toxic to the raised species should be avoided to prevent them from diluting or accumulating in the circulating water. For example, resin acids in softwoods are acutely toxic to salmonids (Oikari et al. 1983; Peng and Roberts 2000). Hardwoods do not contain resin acid and are therefore a more suitable option for RAS applications. Birch woodchips were chosen for this experiment to avoid any harmful effects of resin acids (Fig. 5).

Depending on the wood species and place of growth, woodchips can contain various compounds that are toxic to the raised species, including salmonids, such as resin acids (Oikari et al. 1983), retene (7-isopropyl-1-methylphenantrene) (Billiard et al. 1999; Oikari et al. 2002), or heavy metals (Świetlik et al. 2012). Additionally, organic compounds and nutrients can leach during the first weeks or month after the start-up, depending on operating conditions, as shown in Figs. 4A and 5. This is one of the downsides of woodchip denitrification bioreactors and should already be considered in the process design stage (Healy et al. 2012; Schipper et al. 2010; Christianson et al. 2012; Lepine et al. 2020).

Long-chained unsaturated fatty acids are toxic to salmonids (Leach and Thakore 1978) and are also contained by hardwoods. In this study, several fatty acids were detected in the bioreactors of the side-looped systems (Fig. 5). Octanoic acid has biocidal properties, while hexanoic acid has biocidal and plant protection properties (ECHA 2019). Even benzoic acid is known to have biocidal and corrosive properties, and it is hazardous to health (ECHA 2019). For benzoic acid, an EC50 value (9 mg L^−1^) has been reported in a chronic study with cyanobacterium *Anabaena inaequalis*, and, in another study, researchers reported a 48-h LC50 value of 460 mg L^−1^ for the freshwater fish golden ide *Leuciscus idus* (WHO 2000). In this study, the concentrations remained below these limit values. The highest concentrations were found in the woodchip bioreactor, but they were much lower in the sand filter and in the rearing tanks. In the controls, the concentrations mostly remained below the LOD. This suggests that these compounds may have originated from the woodchips. Moreover, the fatty acids were found at the beginning of the experiment and later decreased to very low levels, similar to those in the controls. This indicates the importance of an efficient and sufficient flushing period before the start-up.

### Anions and off-favors

Birch wood contains micronutrients, including chlorine, originating from the soil in the place of growth and typically occurring in wood as anions in an aqueous solution (Werkelin et al. 2005). The concentrations of readily water-soluble chlorine ions were at first at 20-30 mg L^−1^, but then decreased to a level of about 10 mg L^−1^ (Fig. 4A). However, those of sulfate and phosphate remained more stable (Fig. 4C, D). For example, Werkelin et al. (2005) found 70–110 mg Cl kg^−1^ dw in birch wood (*Betula pubescens*) and 40–330 mg kg^−1^ in birch bark. Woodchips are the most likely source of chlorine in the system, but other sources include fish feed and metabolic products of fish (Turcios and Papenbrock 2014).

The concentrations of off-flavors were at a similar range in the circulating water of both side-looped and control systems (Figs. 2 and 3) and no significant difference (p<0.05) between the systems was found (Supplementary Table S6). The highest levels of both off-flavor compounds were found after the woodchip bioreactor (Fig. 2). In the rearing tanks, the concentrations were mostly below 15 ng L^−1^. Typically, the human sensory thresholds for detection in water range from 15 to 200 ng L^−1^ (GSM) and from 18 to 45 ng L^−1^ (MIB, Persson 1980; Maga 1987), while Young et al. (1996) reported 16 ng L^−1^ for GSM and 15 ng L^−1^ for MIB. This explains the low concentrations found in fish (0.2–0.4 ng g^−1^ MIB and 0.2–0.7 ng g^−1^ GSM after 30 weeks, Fig. 3). The concentrations of both compounds remained below the sensory thresholds for rainbow trout: 0.9 ng g^−1^ for GSM and 0.55 ng g^−1^ for MIB, respectively (Persson 1980; Robertson et al. 2005). Furthermore, there was a significant difference in the concentrations of GSM in fish flesh (p<0.05) between the side-looped and control systems (Fig. 3, Supplementary Table S6). A similar effect was not observed in the case of MIB. It is possible that the GSM-producing bacteria have stopped producing or reduced their production due to certain trace elements leached from the woodchips which could have impacted the growth of bacteria and production of GSM (Schrader et al. 2015).

Denitrification removes nitrogen from the system, resulting in different nitrate levels in side-looped systems than in the controls (Fig. 4B). The effect of low or high nitrate levels on off-flavor production has previously been studied (Schrader et al. 2013), but in that study, no correlation was found. With only a few exceptions (Azaria et al. 2020), the effect of denitrification on off-flavor accumulation has only rarely been a subject of research. According to our knowledge, this was the first experiment to study the concentrations of off-flavors in systems with woodchip bioreactor-based denitrification. However, more research is required to discover if denitrification really has an effect on the concentrations of off-flavors, quantify its extent, and identify the mechanisms behind the phenomenon.

### Trace elements

Trace elements can be transferred to a RAS via feed and inlet water (van Bussel et al. 2014), but they can also leach from pipes or fittings (Davidson et al. 2009). Knowing that, all the pipes, valves, and fittings were made of plastic, thereby preventing them from causing an additional trace element load in the experiment. Such processes with woodchip-based denitrification and a sand filter are both potential sources of trace elements. In this study, the detected concentrations of the selected elements were at low or moderate levels and remained below the chronic exposure limit values for aquatic life (Cd 0.72 μg L^−1^, Ni 52 μg L^−1^, and Pb 3.2 μg L^−1^) set by the US EPA (US EPA 2007b). For example, toxic levels of Zn and Cu can cause sudden mortalities (Wedemeyer 1996). In this case, the number of mortalities remained very low throughout the experiment in both system types and especially in the side-looped systems (Table 2).

At the beginning of the experiment, high concentrations of Mn and Cu were found after the woodchip bioreactor but later decreased to low levels (Fig. 7A, B). Additionally, Ni was found at higher levels (300–400 μg L^−1^) at the end of the experiment (Fig. 7A), but its levels remained below the limit values of acute toxicity for aquatic life (Ni 470 μg L^−1^) set by the US EPA (US EPA 2007b). The results suggest that the nickel was of woodchip origin. It is known that certain elements, including nickel, can be found in birch wood after uptake from the soil (Komanicka et al. 2013). In the case of Mn (Fig. 7A), the concentrations were above the limit values for drinking water (50 μg L^−1^, Council Directive 98/83/EC 1998) at the beginning of the experiment, but then decreased to near the limit value. After the sand filter, all the studied elements decreased to below 20 μg L^−1^ levels after a few weeks of the experiment. Apart from Cu, none of these was found in the rearing tanks. The concentrations of Mn and Ni were 6.2 ± 3.3 μg L^−1^ and 5.7± 2.3 μg L^−1^ in the inlet water from Lake Peurunka, an unlikely source of these elements.

The concentrations of Ca, Co, S, Mg, Na, P, and Zn were below the recommended limit values listed by Davidson et al. (2009). The concentrations of Cu were occasionally above the recommended limit of 30 μg L^−1^ in the side-loop but remained below the limit values in the rearing tank water. Furthermore, the concentrations were lower in the side-looped systems than in the controls (Figs. 6 and 7). This suggests that the main source of Cu originated from the denitrification or the sand filter.

Feed can also be a potential source of metals and other trace elements in the system (Sandor et al. 2001). Low concentrations of the studied trace elements were found in feed (Table 4), which were in the same range as or even lower than those in feed reported by Martins et al. (2011) in a study of the rearing of Nile tilapia *Oreochromis niloticus*. Among the studied elements, only Cu was detected in the tank water. The feed may have been the source of Cu in the rearing tank water and even in the fish flesh (Table 4). Although dozens of μg L^−1^ concentrations (Fig. 7A, B) were detected in the woodchip bioreactor and in the sand filter, they did not lead to an accumulation of trace elements in the fish flesh. This is supported by the similar results in the side-looped systems, as well as in the controls.

Lepine et al. (2020) studied the leaching of metals from the denitrifying woodchip bioreactor outflow filled with white ash (*Fraxinus americana*) or Norway maple (*Acer platanoides*) woodchips. They found that after 3 weeks of operation, the leaching of metals ceased and remained at a low level, below the known concern. In this study, some of the trace elements remained below the LODs throughout the experiment, including Zn (Fig. 6) and Cd (Fig. 7), while others decreased to a low level after the first few weeks of the experiment (e.g., Co, Cu, and Pb in Fig. 7B). The results were similar to those reported by Lindholm-Lehto et al. (2020). Based on the results, we can agree with Lepine et al. (2020) that the flushing of the woodchip bioreactor and sand filter is highly recommended prior to start-up and water reuse.

Generally, the concentrations of selected elements were very low and remained below the limit values of acute toxicity to aquatic life (US EPA 2017). The highest concentrations were observed at the beginning of the experiment, but the levels decreased rapidly to a lower level after the start-up. A stabilization period can therefore be recommended for this type of process before introducing the raised species. The results suggest that the studied elements did not accumulate in the system or in the fish flesh. This is of great importance because none of the trace elements must pose a risk to the raised species or to fish consumers.

## Conclusions

The aim of this experiment was to study the potential risks of reusing RAS outflow water treated with a woodchip bioreactor and sand filter. This was a follow-up study to determine the long-term nitrate removal rate and if any adverse effects occurred in terms of water or fish quality. The results showed that high NO_3_-N removal and denitrification efficiency were achieved in woodchip denitrification, followed by a slow sand filtration of an experimental RAS. The chemical analyses showed that harmful compounds or elements did not accumulate in the system, although higher concentrations were detected at the beginning of the experiment. This highlights the importance of a sufficient flushing period for the woodchip bioreactor before the start-up of the system when aiming for water reuse. The accumulation of trace elements in fish was also studied. Very low concentrations of Cu, Mn, and Ni were detected. They were even lower in the side-looped systems than in the controls, suggesting that these concentrations may have originated from sources other than the woodchips. Additionally, the concentrations of off-flavors (GSM and MIB) were studied in the circulating water and fish flesh. Significantly higher (p<0.05) concentrations of GSM were detected in the fish flesh of the controls than in the side-looped systems, but this was not observed in the case of MIB. According to our knowledge, this was the first trial to directly study the effect of woodchip-based denitrification on the formation of off-flavors. However, the confirmation of a significant difference and a deeper understanding of the phenomenon require further study.

## Supplementary information

ESM 1 (DOCX 187 kb)

## Data Availability

The datasets used and/or analyzed during the current study are available from the corresponding author on reasonable request.
