# Supplementary material for: Efficient water treatment achieved in recirculating aquaculture system using woodchip denitrification and slow sand filtration
Source: Environ Sci Pollut Res Int. 2021 Jul 7;28(46):65333–48. doi: 10.1007/s11356-021-15162-0 (PMC8636402; doi:10.1007/s11356-021-15162-0)
Supplement: Supplementary file 1 — (DOCX 187 kb) [file 11356_2021_15162_MOESM1_ESM.docx]

Supplementary Table S1. Measurements of total ammonium (TAN, mg L^-1^), ammonium (HN_3_-N, mg L^-1^), nitrite (NO_2_-N, mg L^-1^), nitrate (NO_3_-N, mg L^-1^), sulfate (SO_4_^2-^, mg L^-1^), alkalinity (mg L^-1^), and pH in the rearing tank water of controls A1, A2 and side-looped systems A6, A7.

| A1 | | | | | | | |
| --- | --- | --- | --- | --- | --- | --- | --- |
| Week | TAN, mg/L | NH_3_‑N, mg/L | NO_2_-N, mg/L | NO_3_-N, mg/L | SO_4,_ mg/L | Alkalinity, mg/L | pH |
| 1 | 0.64 | 0.0012 | 0.074 | 58.5 | n.a. | 25.2 | 6.84 |
| 2 | 0.71 | 0.0013 | 0.077 | 54.6 | 15 | 22.3 | 6.84 |
| 3 | 0.75 | 0.0016 | 0.087 | 55.8 | 15 | 24.0 | 6.91 |
| 5 | 0.46 | 0.0009 | 0.038 | 50.4 | 14 | 19.5 | 6.87 |
| 7 | 0.52 | 0.0015 | 0.042 | 48.4 | 15 | 20.8 | 7.04 |
| 9 | 0.47 | 0.0013 | 0.040 | 55.5 | 18 | 24.6 | 7.03 |
| 11 | 0.48 | 0.0015 | 0.041 | 46.7 | n.a. | 23.4 | 7.08 |
| 13 | 0.51 | 0.0035 | 0.063 | 65.1 | 19 | 43.1 | 7.42 |
| 15 | 0.52 | 0.0038 | 0.047 | 76.6 | 21 | 56.0 | 7.44 |
| 17 | 0.45 | 0.0015 | 0.048 | 71.6 | 20 | 23.6 | 7.10 |
| 19 | 0.42 | 0.0017 | 0.043 | 60.2 | 18 | 24.9 | 7.19 |
| 21 | 0.38 | 0.0012 | 0.054 | 57.9 | 16 | 23.5 | 7.07 |
| 23 | 0.49 | 0.0016 | 0.047 | 78.8 | 23 | 25.5 | 7.09 |
| 25 | 0.48 | 0.0016 | 0.050 | 88.0 | 24 | 25.1 | 7.11 |

n.a. ‑ not analyzed

| A2 | | | | | | | |
| --- | --- | --- | --- | --- | --- | --- | --- |
| Week | TAN, mg/L | NH_3_‑N, mg/L | NO_2_-N, mg/L | NO_3_-N, mg/L | SO_4,_ mg/L | Alkalinity, mg/L | pH |
| 1 | 0.58 | 0.0016 | 0.053 | 57.4 | n.a. | 24.6 | 6.92 |
| 2 | 0.62 | 0.0013 | 0.067 | 55.8 | 14 | 21.3 | 6.80 |
| 3 | 0.56 | 0.0014 | 0.057 | 54.6 | 15 | 23.1 | 6.90 |
| 5 | 0.53 | 0.0014 | 0.048 | 47.6 | 15 | 20.2 | 6.91 |
| 7 | 0.48 | 0.0013 | 0.041 | 46.6 | 16 | 21.7 | 6.92 |
| 9 | 0.42 | 0.0013 | 0.043 | 39.5 | 18 | 23.5 | 6.97 |
| 11 | 0.39 | 0.0011 | 0.035 | 38.6 | n.a. | 18.6 | 6.95 |
| 13 | 0.44 | 0.0033 | 0.036 | 49.2 | 13 | 42.6 | 7.37 |
| 15 | 0.47 | 0.0051 | 0.041 | 54.8 | 15 | 59.3 | 7.53 |
| 17 | 0.44 | 0.0019 | 0.045 | 50.8 | 15 | 26.3 | 7.12 |
| 19 | 0.33 | 0.0014 | 0.037 | 42.8 | 12 | 18.8 | 7.11 |
| 21 | 0.30 | 0.0004 | 0.031 | 36.8 | 10 | 10.7 | 6.63 |
| 23 | 0.42 | 0.0008 | 0.030 | 42.9 | 13 | 14.4 | 6.75 |
| 25 | 0.40 | 0.0007 | 0.028 | 47.4 | 14 | 11.6 | 6.76 |

n.a. ‑ not analyzed

| A6 | | | | | | | |
| --- | --- | --- | --- | --- | --- | --- | --- |
| Week | TAN, mg/L | NH_3_‑N, mg/L | NO_2_-N, mg/L | NO_3_-N, mg/L | SO_4,_ mg/L | Alkalinity, mg/L | pH |
| 1 | 0.66 | 0.0012 | 0.115 | 32.7 | n.a. | 28.4 | 6.82 |
| 2 | 0.74 | 0.0011 | 0.054 | 30.4 | 9 | 24.9 | 6.73 |
| 3 | 0.68 | 0.0012 | 0.054 | 33.6 | 11 | 22.2 | 6.83 |
| 5 | 0.68 | 0.0012 | 0.039 | 27.6 | 14 | 21.8 | 6.81 |
| 7 | 0.76 | 0.0014 | 0.038 | 31.8 | 14 | 21.2 | 6.85 |
| 9 | 0.60 | 0.0015 | 0.034 | 36.7 | 14 | 21.9 | 6.99 |
| 11 | 0.44 | 0.0012 | 0.021 | 26.5 | n.a. | 19.5 | 7.03 |
| 13 | 0.51 | 0.0010 | 0.024 | 35.7 | 12 | 20.5 | 6.88 |
| 15 | 0.51 | 0.0021 | 0.033 | 43.0 | 14 | 24.2 | 7.19 |
| 17 | 0.52 | 0.0019 | 0.040 | 40.4 | 17 | 24.0 | 7.13 |
| 19 | 0.40 | 0.0021 | 0.035 | 34.0 | 15 | 24.8 | 7.29 |
| 21 | 0.33 | 0.0017 | 0.025 | 28.4 | 13 | 26.8 | 7.30 |
| 23 | 0.38 | 0.0034 | 0.011 | 27.9 | 13 | 49.7 | 7.53 |
| 25 | 0.42 | 0.0036 | 0.020 | 38.0 | 16 | 50.4 | 7.51 |

n.a. ‑ not analyzed

| A7 | | | | | | | |
| --- | --- | --- | --- | --- | --- | --- | --- |
| Week | TAN, mg/L | NH_3_‑N, mg/L | NO_2_-N, mg/L | NO_3_-N, mg/L | SO_4,_ mg/L | Alkalinity, mg/L | pH |
| 1 | 0.99 | 0.0621 | 0.201 | 47.0 | n.a. | n.a. | 8.06 |
| 2 | 0.82 | 0.0324 | 0.074 | 42.8 | 13 | 213.1 | 7.85 |
| 3 | 0.83 | 0.0175 | 0.059 | 31.6 | 12 | 103.4 | 7.57 |
| 5 | 0.77 | 0.0061 | 0.043 | 29.9 | 12 | 42.5 | 7.14 |
| 7 | 0.88 | 0.0050 | 0.050 | 33.4 | 13 | 28.4 | 7.00 |
| 9 | 0.66 | 0.0035 | 0.040 | 36.8 | 16 | 26.4 | 6.97 |
| 11 | 0.58 | 0.0028 | 0.035 | 31.0 | n.a. | 22.1 | 6.92 |
| 13 | 0.56 | 0.0033 | 0.034 | 46.3 | 15 | 24.8 | 7.01 |
| 15 | 0.60 | 0.0053 | 0.041 | 52.6 | 22 | 28.4 | 7.19 |
| 17 | 0.62 | 0.0040 | 0.041 | 49.8 | 24 | 26.8 | 7.05 |
| 19 | 0.71 | 0.0059 | 0.048 | 45.4 | 23 | 25.5 | 7.16 |
| 21 | 0.52 | 0.050 | 0.039 | 39.0 | 21 | 30.1 | 7.23 |
| 23 | 0.58 | 0.0234 | 0.030 | 43.5 | 24 | 130.0 | 7.86 |
| 25 | 0.56 | 0.0322 | 0.025 | 49.0 | 29 | 175.6 | 8.02 |

n.a. ‑ not analyzed

Supplementary Table S2. Level of detection (LOD), level of quantification (LOQ), and linearity (R^2^) of selected standard solutions (1‑100 mg L^-1^) for IC analysis.

| **Anion** | **LOD** | **LOQ** | **Linearity, R^2^** |
| --- | --- | --- | --- |
| Cl^‑^, mg L^-1^ | 0.093 | 0.176 | 0.9996 |
| NO_2_^‑^, mg L^-1^ | 0.365 | 0.476 | 0.9973 |
| NO_3_^‑^, mg L^-1^ | 0.219 | 0.243 | 0.9980 |
| SO_4_^2‑^, mg L^-1^ | 1.042 | 1.157 | 0.9988 |
| PO_4_^3‑^, mg L^-1^ | 0.097 | 0.102 | 0.9991 |

Supplementary Table S3. Instrumental parameters and measurement conditions for Perkin Elmer NexION 350 D ICP‑MS spectrometer.

| Isotopes monitored | Cd^111^, Cd^112^, Cd^114^, Co^59^, Cu^63^, Cu^65^, Mn^55^, Ni^58^, Ni^60^, Pb^206^, Pb^207^, Pb^208^ |
| --- | --- |
| Spray chamber | Cyclonic |
| RF power | 1600 W |
| Plasma gas flow rate | 18 L min^-1^ |
| Nebulizer | PFA-ST |
| Ar nebulizer gas flow rate | 0.85-0.9 L min^-1^ |
| Injector | Perkin Elmer 1.8 mm I.D. Sapphire |
| Injection volume | 1.5 mL |
| Sampling cone | Ni, 1 mm aperture diameter |
| Skimmer cone | Ni, 0.4 mm aperture diameter |
| Scan mode | peak hopping |
| Dwell time | 50 s |
| Sweeps per reading | 24 |
| Integration time | 1200 ms |
| Readings per replicate | 3 |

Supplementary Table S4. Level of detection (LOD), level of quantification (LOQ), and linearity (R^2^), listed for cadmium (Cd^111^), cobalt (Co^59^), copper (Cu^63^), manganese (Mn^55^), nickel (Ni^60^), and lead (Pb^206^), µg L^-1^ for ICP‑MS analysis.

| **Analyte** | **LOD (µg L^-1^)** | **LOQ (µg L^-1^)** | **R^2^** |
| --- | --- | --- | --- |
| Cd^111^ | 0.12 | 0.40 | 0.9999 |
| Co^59^ | 0.27 | 0.92 | 0.9997 |
| Cu^63^ | 0.02 | 0.07 | 0.9999 |
| Mn^55^ | 0.03 | 0.11 | 0.9999 |
| Ni^60^ | 0.15 | 0.50 | 0.9999 |
| Pb^206^ | 0.06 | 0.22 | 0.9999 |

Supplementary Table S5. Level of detection (LOD), quantification (LOQ), and linearity (R^2^), listed for aluminum (Al), calcium (Ca), iron (Fe), potassium (K), magnesium (Mg), sodium (Na), phosphorous (P), sulfur (S), and zinc (Zn), mg L^-1^ for ICP‑OES analysis.

| **Analyte (nm)** | **LOD (mg L^-1^)** | **LOQ (mg L^-1^)** | **R^2^** |
| --- | --- | --- | --- |
| Al (396.153) | 0.148 | 0.36 | 0.9998 |
| Ca (393.366) | 0.49 | 2.20 | 0.9999 |
| Fe (259.939) | 0.06 | 0.20 | 0.9999 |
| K (766.490) | 3.02 | 8.74 | 0.9996 |
| Mg (279.077) | 0.28 | 0.94 | 0.9999 |
| Na (589.592) | 0.01 | 0.07 | 0.9999 |
| P (214.914) | 0.22 | 0.89 | 0.9999 |
| S (181.975) | 0.13 | 0.63 | 0.9999 |
| Zn (206.200) | 0.01 | 0.10 | 0.9999 |

Supplementary Table S6. Independent samples t-test of GSM and MIB in circulating water and in fish flesh, experiment vs. control at 95 % confidence interval (p=0.05).

| **Tested:** **experiment vs. control** | **Mean difference** | **95 % confidence interval of the difference** | **p** | **significance** |
| --- | --- | --- | --- | --- |
| MIB in fish after 23 weeks, ng g^-1^ | 0.083 | -0.088 0.253 | 0.282 | not significant |
| MIB in fish after 30 weeks, ng g^-1^ | 0.140 | -0.194 0.474 | 0.345 | not significant |
| GSM in fish after 23 weeks, ng g^-1^ | 0.350 | 0.054 0.646 | 0.027 | significant |
| GSM in fish after 30 weeks, ng g^-1^ | 0.493 | 0.190 0.797 | 0.009 | significant |
| MIB in water, ng L^-1^ | 1.603 | -1.259 4.464 | 0.262 | not significant |
| GSM in water, ng L^-1^ | -1.620 | -5.087 1.847 | 0.347 | not significant |

Supplementary Table S7. Independent samples t-test of selected elements in the tank water, experiment vs. control at 95 % confidence interval (p=0.05).

| **Tested: experiment vs. control** | **Mean difference** | **95 % confidence interval of the difference** | **p** | **significance** |
| --- | --- | --- | --- | --- |
| Al, mg L^-1^ | 0.164 | -0.166 0.494 | 0.324 | not significant |
| Ca, mg L^-1^ | -0.923 | -1.514 -0,332 | 0.003 | significant |
| Cu, µg L^-1^ | 5.789 | 1.918 9.659 | 0.004 | significant |
| Fe, mg L^-1^ | -0.034 | -0.066 -0.002 | 0.040 | significant |
| K, mg L^-1^ | -3.032 | -5.740 -0.324 | 0.029 | significant |
| Mg, mg L^-1^ | -0.016 | -0.179 0.148 | 0.850 | not significant |
| Na, mg L^-1^ | 16.775 | 1.308 32.241 | 0.034 | significant |
| P, mg L^-1^ | 0.329 | -0.253 0.911 | 0.262 | not significant |
| S, mg L^-1^ | -0.029 | -1.014 0.957 | 0.954 | not significant |
| Zn, mg L^-1^ | 0.007 | -0.0004 0.015 | 0.062 | not significant |


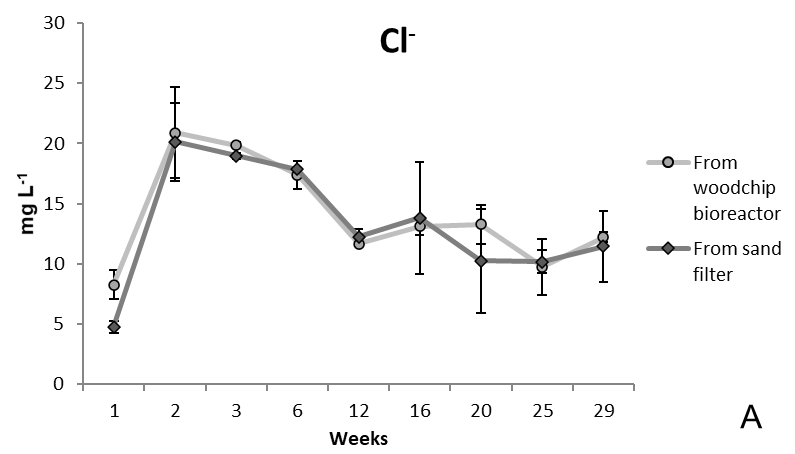

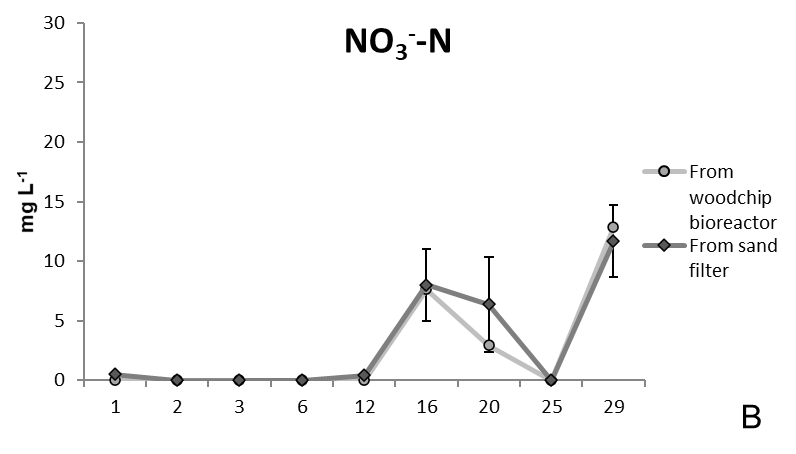


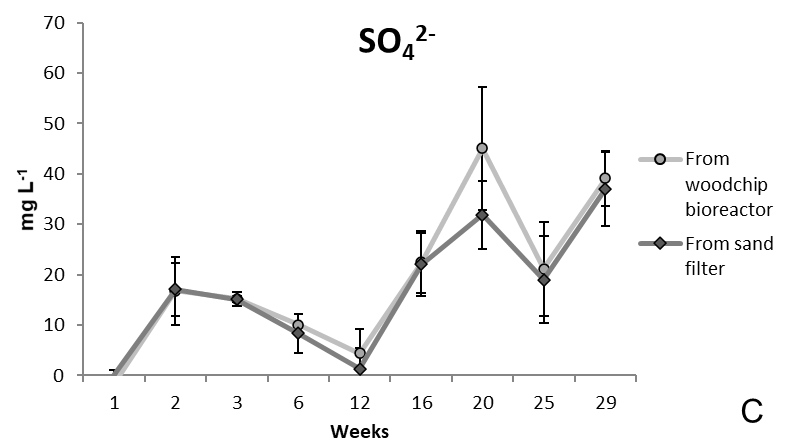

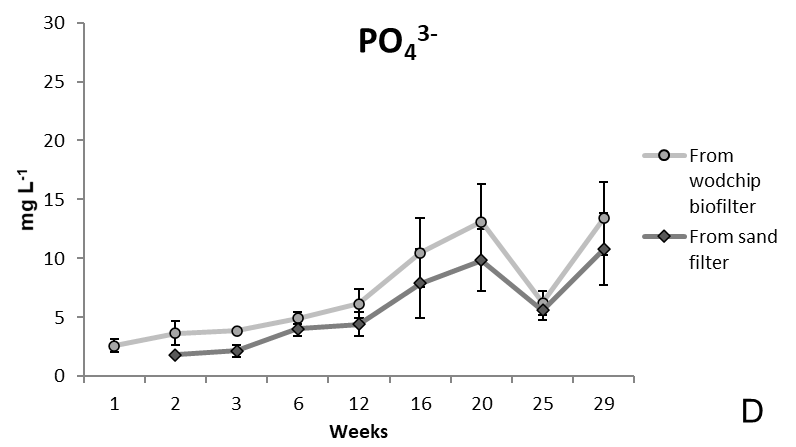


Supplementary Fig. S1. Concentrations of chlorine (Cl^-^, A), nitrate-N (NO_3_^-^-N, B), sulfate (SO_4_^2-^, C), and phosphate (PO_4_^3-^, D) in the circulating water in the woodchip bioreactor and in the sand filter during the 30 weeks of the experiment (mg L^-1^, ± SD, n=4).


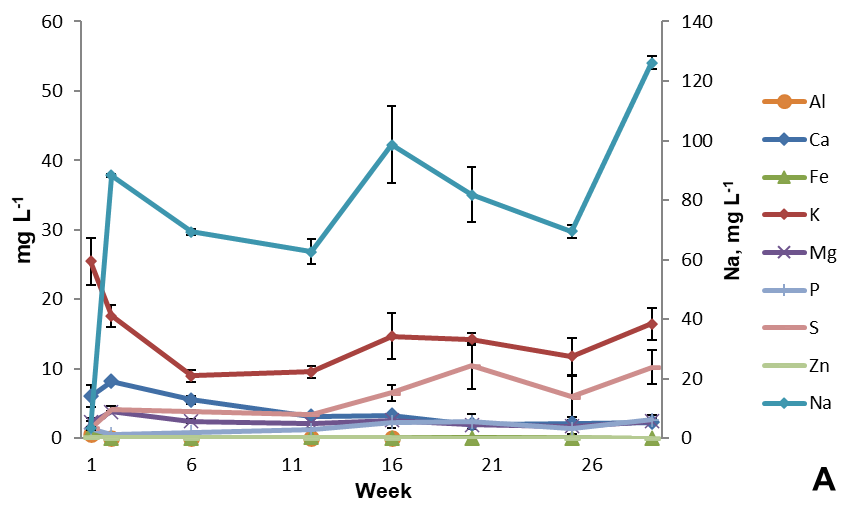


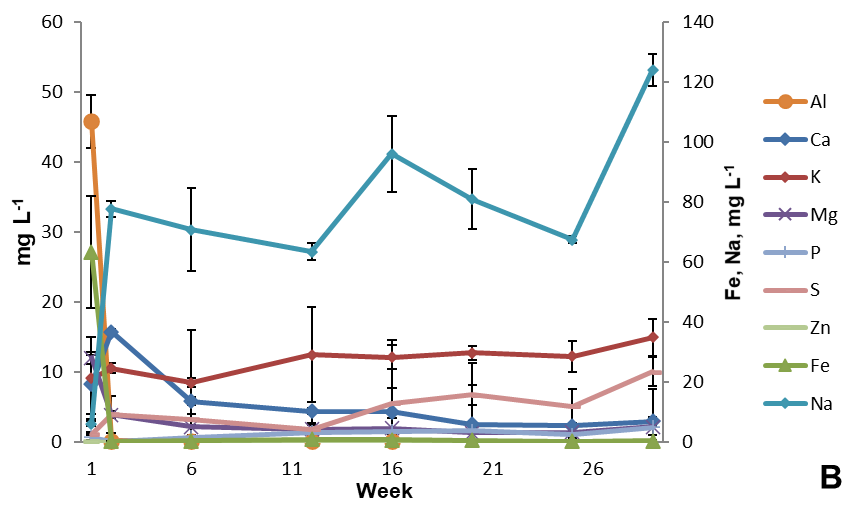


Supplementary Fig. S2. Concentrations of aluminum (Al), calcium (Ca), iron (Fe), potassium (K), magnesium (Mg), phosphorous (P), sulfur (S), zinc (Zn), and sodium (Na, axis on the right hand side) after the woodchip bioreactor (A) and after the sand filter (B, Fe and Na axis on the right hand side) during the 30 weeks of the experiment (mg L^-1^ ± SD, n=4).
